# The relationship between joint mobility and motor performance in children with and without the diagnosis of developmental coordination disorder

**DOI:** 10.1186/1471-2431-13-35

**Published:** 2013-03-15

**Authors:** Lemke D Jelsma, Reint H Geuze, Mariette H Klerks, Anuschka S Niemeijer, Bouwien CM Smits-Engelsman

**Affiliations:** 1 Developmental and Clinical Neuropsychology, University of Groningen, Groningen, The Netherlands; 2 Faculty of Kinesiology and Rehabilitation Sciences, KU, Leuven, Belgium; 3 Avans+, Breda, The Netherlands; 4 Practice for PT, De Pol 5A, Peize, VA, 9321, The Netherlands; 5 Association of Dutch Burn Centers, Burn Centre, Martini Hospital Groningen, Groningen, The Netherlands

**Keywords:** Beighton score, Hypermobility, Motor performance, Movement assessment battery for children, DCD, Children

## Abstract

**Background:**

The purpose of this study was to determine whether joint mobility is associated with motor performance in children referred for Developmental Coordination Disorder (DCD-group) in contrast to a randomly selected group of children between 3–16 years of age (Random-Group).

**Methods:**

36 children with DCD and 352 typically developing children (Random-Group) participated. Hypermobility was classified based on the Beighton score (cut-off ≥5 for 3–9 years and ≥4 for 10–16 years) using goniometry. Motor performance was assessed with the Movement Assessment Battery for Children (MABC).

**Results:**

The mean Beighton score in the DCD-group was 5.0 versus 2.6 in the Random group. Prevalence of hypermobility was higher in the DCD-group than in the Random Group (64% and 33% respectively; χ ^2^ = 16.09, *p* < .001). There was a significant [negative] correlation (r_p_ = −.38, *p* = .02) between Beighton score and total MABC scores within the DCD group, but not in the Random Group (r_p_ = −0.07, *p* = .20). More specifically, in the DCD group we found a significant negative correlation between the MABC total score and the degree of hyperextension of the knees.

**Conclusion:**

The extremely high prevalence of hypermobility when applying the recommended cut-off scores stresses the need for an international agreement on firm cut-off points and the use of standardized measurement of Beighton mobility manoeuvres. The results of this study show that a cut-off of 7 is more appropriate, resulting in a prevalence of 6% in children aged 3–16 years. Although in the general population motor performance and joint mobility are not related, this is the case in children referred for DCD. We argue that more mobility of the joints may be a disadvantage when motor coordination is poorly developed.

## Background

Generalized hypermobility is said to be present when many joints in the body show an increased range of motion [1]. Most children with hypermobility have no complaints and are referred to as children with asymptomatic generalized hypermobility [2]. Some children even benefit from excessive range of motion in their joints to succeed in gymnastic or acrobatic skills. Middleditch [3] however describes the difference between ballet dancers and gymnasts with ‘normal tissues’ and increased range of motion due to training and stretching versus hypermobile individuals with generally weaker tissue. This tissue is less resilient; joints are less stable and vulnerable to subluxation. Hypermobile ballet dancers have a higher incidence of injuries than non-hypermobile dancers [4]. The prevalence of asymptomatic generalized hypermobility in children has been variably and widely reported, between 3% and 30% [5-7]. Hypermobility is generally measured with the Beighton test [2,8]. Importantly, there seems a strong association between disorders of hypermobility and Developmental Coordination Disorder (DCD) [9].

Children diagnosed with DCD show levels of motor coordination below that expected for their chronological age and intelligence. These coordination deficiencies lead to problems in activities of daily living as well as in academic performance [10]. Maillard and Murray hypothesized that clumsiness, reported by parents of children with hypermobility, might be due to reduced proprioception from the joints [11]. This in combination with suboptimal strength of the muscles will then lead to poor control of joint movement and instability. The literature about the relation between hypermobility and motor performance is inconsistent (Table 1). Engelbert et al. conducted a retrospective study (n = 72) in which two groups of children with hypermobility were studied; a very young group (1–2,5 years) and an older group (4–12 years) [12]. Although considerable percentages (of 56 and 25 respectively) were found to have a delay in motor development, no association was found between the Bulbena score as measure for hypermobility and the Bayley Scales of Infant Development or the Movement Assessment Battery for Children (MABC) as measure of motor performance. It was however assumed that generalized hypermobility may influence motor development in children, since the median score of the hypermobile group was around the 15th percentile on the MABC (Table 1) [13]. In a combined retrospective and prospective study of children with hypermobility referred to a rheumatology clinic, Adib et al. [7] used a questionnaire and found that 48% of hypermobile children were also considered clumsy, 36% had poor coordination and 7% was diagnosed as having dyspraxia.

**Table 1:** Characteristics of individual studies of both hypermobility and DCD

**Study**	**Type of Sample**	**N**	**Measure Instrument**	**Outcome (%)**
Adib et al. [7]	Prospectively (1) through hypermobility clinic and retrospectively (2) through rheumatology department included, based on referrals by specialists or hospital notes*	125	(1) Beighton score	Clumsy 44/92 (48%)
			Medical examination	Easy bruising 39/91(43%)
			(1 &2) Questionnaire	Poor coordination 30/86 (36%)
				Walked after 15 months 19/57(33%)
				Clicky joints 25/84(30%)
			Age range3-17 years	Learning difficulty 13/91 (14%)
	*number per group unknown			Dyslexia 2/88 (2%)
				Dyspraxia 6/87 (7%)
Engelbert et al. [12]	Retrospective hospital chart review of children with hypermobility complaints		Bulbena: passive maneuvers of 9 joints (thumb, little finger, elbow, shoulder, hip, knee, patella, ankle, and first metatarsophalangeal joint) and the presence of ecchymoses is recorded. Generalized hypermobility of the joints is present when a score > =5 is obtained in females and > =4 in males.	Delay in motor development:
				(1) 9/16 (56%), no significant association between the delay in motor development (yes/no) and the Bulbena score.
	n = 16 < 2.5 years of age (1)	16		
	n = 56 ≥ 4 years of age (2)	56		
				(2) severe; 14/56 (25%), at risk 12/56 (21%), age appropriate 30/56 (54%). No significant association between delay in motor development and the Bulbena score found. Median score P15
			1-2.5 years: Bayley Scales of Infant Development	
			4-12 years: Movement Assessment Battery for Children	
Kirby and Davies [14]	Random group (mean age 10.8y, range 5-18y) of TDC (1)	27	(1)Parental responses and Movement ABC Checklist.	37% of the children with DCD against 7.4% in the TDC group had symptoms of JHS.
			(2)MABC score <5%	
	Children diagnosed with DCD (mean age 12.5y, range 9-17y) (2)	27	(1)& (2) A questionnaire based on the ‘five-part questionnaire for identifying hypermobility’	
Hands and Larkin [16]	Children with motor learning difficulties (MLD)	52	McCarron Assessment of neuromuscular development (MAND 1982) MABC (1992)	Overall, the group with MLD was significantly less flexible than the control group. The group with MLD had a higher BMI and lower performance levels on the sit and reach, sit-ups, standing broad jump, 50-metre run, and the shuttle run.
	Age and gender matched control group	52		
			Qualitative observation of poor motor performance skills	
			Fitness assessment battery	
Cantell et al. [15]	Children (8–9 years), adolescents (17–18 years) and adults (20–60 years) with high or low motor performance	39	MABC	The low motor competence groups scored higher on the BMI, had a greater percentage of body fat and showed poorer fitness results in endurance, flexibility and strength
		44	DCDQ	
		66	Body Composition	
			Cardio Respiratoir Fitness	
			Flexibility	
			Muscle strength	
			Lung capacity	
			Leisure participation	

However, since in these both studies the initial entry was hypermobility, none of the studies mentioned neither yielded significant evidence for co-occurrence of hypermobility and poor motor performance in children referred for motor problems or for children in a general nonclinical population. Kirby and Davies [14] did look at the co-occurrence in the other direction. They compared children diagnosed with DCD (n = 27) and a typically developing group (TDC; n = 27) using a questionnaire evaluating the clinical signs of children with hypermobility. Thirty-seven per cent of the children with DCD had symptoms of hypermobility (JHS), compared with 7,4% in the TDC group. Signs such as bending the thumb back to touch the forearm, contorting the body into strange postures or being double jointed were considerably more prevalent in the group of children with DCD compared to their matched peers.

Another approach used is looking into the physical fitness of children with high and low motor competence [15]. One of the items used to assess physical fitness and flexibility of the hip joints, lower back and length of Hamstrings, was the sit and reach test. The group with low motor competence had poorer results in flexibility. This finding is supported by Hands et al. (2006) in children with motor learning difficulties, while Schott et al. did not find a relationship with flexibility, measured with the sit and reach test, in a group of children with DCD [16,17].

It can be concluded that there is evidence (in retrospective or questionnaire studies) of a higher incidence of motor delay in children who are referred for hypermobility, even in the absence of an identified neurological deficit. There is also evidence of enhanced flexibility in children with DCD [12,18,19]. Since humans have a complex body with more joints and with that more degrees of freedom than needed to perform any particular task, a flexible and adaptable motor system is needed to control all joints in a given task [20]. A limited capacity to control the degrees of freedom, as seen in children with DCD, shows up as inefficient timing of antagonist activation resulting in prolonged movement time and a need for more corrective movements during target directed motion [21]. This may be one of the underlying causes of the development of poor postural control and movement skills [9]. Since hypermobility results in larger degrees of freedom of motion in joints, it is important to better understand the consequences of hypermobility and its related symptoms during the motor development of both typically developing children and those with DCD.

The present study was designed to elucidate the association between joint mobility and motor performance. To estimate range of motion (ROM) goniometry was used to objectively measure joint mobility. The first aim of the study was to verify whether children referred for DCD would present more often with hypermobility (as measured by the Beighton score) than children in a random sample [2,8]. A second aim was to evaluate the relationship between motor performance (as measured by the MABC) and joint mobility in a random sample of children aged 3–16 years old, without medical or neurological complaint [13,22]. Finally we tested whether the relation between mobility and motor performance was different between the children with poor motor coordination and the general-population sample. Based on the literature, it was hypothesized that hypermobility would be associated with lower motor performance in both groups, but to different degrees, with a stronger association in the DCD group.

## Methods

### Participants

Children with DCD referred to a rehabilitation centre or paediatric physical therapists for their motor problems were asked to participate before they were given treatment (DCD-group). Children were included when motor coordination problems were interfering with daily activities at school or home. A general-population sample of children attending mainstream (pre)education was randomly selected by choosing every second child on the class list of the participating schools (Random-group). Children were excluded if they were in need of special care or medication, or had limitations due to cardiopulmonary, neurological, rheumatic, orthopaedic or metabolic conditions, or had an IQ < 70. Following the reasoning by Geuze et al. children in regular primary schools may be considered to have an IQ within the normal range [23].

The DCD-group was age-matched with children from the random sample (henceforth called matched Typically Developing or matched TD-group).

### Instruments

#### The Beighton hypermobility score

The Beighton hypermobility score is composed of five manoeuvres (see Table 2) [2,8]. Four of them are tested passively on both sides of the body and one is actively tested. The passive extension of the metacarpophalangeal joint of the little finger (MCP5), elbow and knee were measured bilaterally according to a standardized joint mobility protocol [24]. When the ROM exceeds a specified range one point is given. These points are summed and the score ranges from zero to nine (two times four joints and one point for hands flat on the floor with straight knees). According to the recommendation of van der Giessen the cut-off point for hypermobility was ≥ 5 for children aged 3–9 years old and ≥ 4 points for children aged older than 10 years [25]. Children at or above the cut-off score were henceforth classified as hypermobile, children below this score as typically mobile.

**Table 2:** **The nine-point Beighton hypermobility score**[2,8]

**The ability to:**	**Right**		**Left**
1. Passively dorsiflex the fifth metacarpophalangeal joint to ≥ 90°	1		1
2. Passively oppose the thumb to the volar aspect of the forearm	1		1
3. Passively hyperextend the elbow to ≥ 10°	1		1
4. Passively hyperextend the knee to ≥ 10°s	1		1
5. Actively place hands flat on the floor without bending the knees		1	
Total	9 points		

One point may be gained for each side for manoeuvres 1–4 so that the hypermobility score will have a maximum of nine points if all are positive.

The Beighton hypermobility score is considered to be a valid instrument for children [24,25]. Goniometry is recommended for measuring the degrees of movement accurately. A Collehon Extendable Goniometer type 01135 (Lafayette instruments) was used for the passive measurement of the knee and elbow. A smaller goniometer type HIRes (Baseline CE) was used to measure the mobility of the little finger. In the present study the same standardized joint mobility protocol was used as in the study by Smits-Engelsman et al. [24]. Reliability for this protocol was high (ICC = 0.99) [24].

#### The Movement Assessment Battery for Children

The MABC 1st (n = 117) and 2nd editions (n = 271) were used to test the children’s motor performance, since the data collection took place before and during the introduction of the second edition in the Netherlands [13,22,26]. The MABC is a standardised and norm referenced test and validated for the Dutch population [22]. The aim of the MABC is to classify children according to degree of motor impairment. There are separate age-related item-sets, each consisting of 8 items measuring manual dexterity (3 items), aiming and catching (2 items), and balance (3 items). Total scores can be transformed into percentiles. The structure of the two versions of the MABC and the content of most items are similar, therefore percentile scores are considered to be comparable. In order to use the outcomes of both editions of the test, all motor scores were transformed to percentiles. A percentile score of 6–15 is considered to be indicative of a risk of motor problems whereas a score ≤ the 5th percentile is indicative of a serious motor problem [13,26]. Both editions of the MABC have been shown to be valid instruments to measure motor performance for this age-group [22,26-30]. Test-retest reliability for both editions is also similarly good (ICC’s range from 0.95-0.98) [22,26].

### Procedure

The three year old children were tested at home; the older children were tested at school in a quiet room; the DCD-group was tested at a rehabilitation centre or in the private practice known to the child. Each session, carried out by the same tester, started with the MABC and finished with the nine manoeuvres for the Beighton score, of which six were tested once with goniometry. The time needed to test a child was on average 45 minutes. All tests were administered individually by one of five trained paediatric physical therapists. These specialised therapists have all passed a criterion test for measurement reliability for both the Beighton score and the MABC.

### Ethics

The Independent Review Board (METC Amsterdam, nr. 06.0517/NL11694.003.06) gave its approval to the study. Parents gave their written informed consent.

### Data analysis

Descriptive statistics were used for the characteristics of the children and to calculate frequency, percentage, medians, mean and standard deviation (SD) of the outcome variables. As mentioned, the classification for typically mobile and hypermobile was corrected for age by using different cut-off values. Chi-Squared test was performed to test for differences in the frequency of hypermobility in the Random-group and DCD-group. However, the scores for maximum joint mobility measured by goniometry of knee, elbow and little finger could not be corrected for age because no age norms are available. Therefore the DCD-group was age-matched with 36 children from the random sample (matched TD-group). T-tests were performed to test for differences in Beighton score and degrees of maximum joint mobility between the DCD-group and matched TD-group. Relations between the Beighton score and the MABC percentiles were calculated within the matched TD group as well as the DCD group using Spearman rank correlation. Relations between ROM and MABC percentiles within the DCD group and within the matched TD group were calculated using Pearson correlations. A correlation of .75 and above was considered good, those between .75 to .50, moderate, and those below .50, poor [31]. An alpha level of 5% was adopted for all analysis.

## Results

### Participant data

In total 388 children from the Netherlands participated in this study. For the DCD group a total of 64 children were invited through PT practices to participate in the study and 36 agreed (56% response). The DCD-group had a mean age of 8.0 years, with a range between 7–10 years and consisted of 27 boys (75%) and nine girls (25%). One child scored at the 25th percentile of the MABC, but was included because DCD had been diagnosed at the Rehabilitation Centre.

A general-population sample of 352 children attending mainstream (pre)education was included for the Random-group. The Random-group had a mean age of 10.6 years with a range between 3–16 years (SD 4.3) and consisted of 169 boys (48%) and 183 girls (52%).

The DCD-group was age-matched with 36 children from the random sample, with also a mean age of 8.0 years and a range between 7–10 years, consisting of 20 boys (56%) and 16 girls (44%) (matched TD-group).

### Group differences in motor performance

The median MABC percentile of the DCD group was 2 (mean 4.07; SD 5.0, Range 0–25). The thirty children of the DCD group scored at or below the 5th, five between the 5th and 16th and one (with a definite diagnosis of DCD) at 25th percentile. The median MABC percentile of the Random-group was 75 (mean 69.2; SD 25.6, range 3–100).

### Frequency of hypermobility and differences between groups

Mean Beighton score of the DCD-group was 5.0 (SD 2.3) and the percentage of hypermobility was 64% (n = 24), while these values were 2.9 (SD 2.2) and 33% (n = 116) for the Random-group of children. The percentage of children classified as hypermobile differed significantly between the DCD group and the Random-group (*χ*^2^ = 16.09, *p* < .001).

As expected, the matched TD-group and the DCD group also differed on the Beighton score (Table 3). As previously mentioned the DCD group had a mean Beighton score of 5.0 (CI 4.2-6.0) while the TD-group had a mean Beighton score of 3.6 (CI 2.9-4.2) and a hypermobility percentage of 33% (n = 12). The number of children that could oppose the thumb to forearm and the number that could get their hands on the floor with straight legs did not differ between the matched TD-group and DCD-group (respectively right thumb *χ*^2^ = 0.22, *p* = .64; left thumb *χ*^2^ = 0.90, *p* = .34 and hands on floor *χ*^2^ = 1.86, *p* = .17). Moreover, the ranges of motion of the DCD-group were larger than those for the matched TD-group in the little finger and in the knee but not in the elbow with *p*-values between < .001 to .013 (Table 3).

**Table 3:** **Mean (SD), *t *- and *p *-values for joint mobility in the matched Typically Developing-group (n** **= 36) and DCD-group (n** **= 36)**

**Mobility**	**Mean (SD) matched TD-group**	**Mean (SD) DCD-group**	***t*-value**	***p-*value**
Beighton score	3.6 (1.9)	5.0 (2.3)	−2.73	.008
Knee Left #	6.9 (3.7)	11.3 (4.8)	−4.42	<.001
Knee Right #	6.8 (4.3)	11.2 (4.7)	−4.15	<.001
Elbow Left #	14.0 (3.5)	12.7 (6.6)	1.09	.278
Elbow Right #	14.3 (4.4)	12.6 (5.8)	1.33	.189
MCP5 Left #	88.2 (6.9)	94.9 (12.6)	−2.76	.007
MCP5 Right #	87.1 (9.2)	93.9 (13.0)	−2.55	.013

# Maximum joint extension in degrees per joint.

*MCP5* Meta Carpo Phalangeal Joint of the 5th finger.

### Relation between overall mobility and motor performance

The correlation within the Random-group and within the matched TD group between the Beighton score and MABC was virtually absent (r_s_ = −0.07, *p* = .20, r_s_ = −0.04, *p* = .82 respectively). However the correlation within the DCD-group between the Beighton and MABC score was significant though poor, with explained variance of 0.14 (r_s_ = −0.38, *p* = .02). Table 4 lists the Pearson correlations between range of motion and the MABC percentile within the DCD group and matched TD group. There was a significant negative moderately poor correlation between the range of motion in the knee joints and the MABC percentile within the DCD group in contrast to the matched TD group (left r_s_ = −0.48, *p <* .01; right r_s_ = −0.37, *p* = .03).

**Table 4:** Pearson correlations between MABC percentile score and joint mobility measures within the DCD-group and matched TD group

**Pearson correlation**	**DCD group (sign. 2-tailed)**	**Matched TD group**
MCP5 Left	**−**.29 (.087)	.21 (.211)
MCP5 Right	**−**.20 (.246)	.06 (.729)
Elbow Left	**−**.31 (.068)	.31 (.063)
Elbow Right	**−**.31 (.063)	.22 (.201)
Knee Left	**−.48 (.003)**	.01 (.967)
Knee Right	**−.37 (.025)**	.05 (.778)

*MCP5* Meta Carpo Phalangeal Joint of the 5th finger.

## Discussion

The aim of this study was to determine the prevalence of hypermobility in children with Developmental Coordination Disorder and in a randomly selected group of typically developing children in a wide age range (3–16 years of age). It was investigated whether hypermobility and ROM were associated with motor performance in both groups and whether the relation was different between the DCD group in contrast to an age matched group. For this purpose a sample of 36 children referred for DCD to physical therapy intervention and a random sample of 352 typically developing Dutch children was examined. They were assessed with the MABC to establish their motor performance and for joint mobility with goniometry to establish an objective value for the Beighton score.

It is of interest that the prevalence of hypermobility was twice as high in children with DCD (64%) as compared with the prevalence of hypermobility in both the random and matched TD groups, which was also rather high (33%). These percentages were obtained using the cut-off points for hypermobility recommended by van der Giessen [25], which were more strict than those reported by Beighton [2,8]. Nevertheless, the high prevalence of hypermobility found in our study of typically developing children is difficult to accept and is much higher than the one reported by Jessee et al., who reported 5-7% of school children to be hypermobile [32]. In their study of Swedish school children, Jansson et al. stated that if hypermobility is considered a variation of general joint laxity such a high prevalence of non-normality is difficult to accept [6]. Our data substantiate that statement. The findings of this study also corroborate with the recommendation of Smits-Engelsman et al. to choose the 7 on the 9 point Beighton scale as the cut-off in children over 6 years of age, when mobility is tested passively by goniometry [24]. This raises the question whether the prevalence in both groups is more acceptable with a cut-off of 7. If a 7-point cut-off is applied to the data in the present study, the prevalence of hypermobility would become 6% in the general population, which is regarded as an acceptable percentage. For the DCD-group the prevalence would be 28% if this higher cut-off were used. A posthoc analysis shows that with the new criterion within the DCD group the subgroups with (n = 10) and without (n = 26) hypermobility clearly differ on the MABC2 (mean score 2.8 (SD = 1.8) versus 4.4 (SD = 1.7), *t*(2,34) = 2.5, *p* = .019). So far there remains uncertainty in the literature about cut-off points and ways to measure hypermobility. We therefore strongly recommend an international agreement on cut-off points and the use of the standardized measurement of Beighton mobility manoeuvres, since the prevalence of hypermobility in both random and clinical populations is otherwise difficult to compare.

Regardless of the chosen cut-off scores the prevalence of hypermobility, at 28%, was proportionally still much higher in the group of children referred for DCD (*χ*^2^ = 17.93, *p* = .001). Based on the literature, it was anticipated that asymptomatic joint hypermobility would be associated with poor motor performance. Interestingly, this was not the case in the randomly selected group (r_p_ = −.07). Given results in the random group, there seems no association between joint mobility and motor performance among typically developing children. However, the negative correlation within the DCD-group was significant and moderately poor (r_p_ = −0.38, *p* = .02). These findings demonstrate that when poor motor performance occurs, especially in tasks dealing with control of the whole body, 14% of the performance can be explained by asymptomatic joint hypermobility (*R*^*2*^ = 0.14). Our findings provide evidence that testing for hypermobility, preferably by a standardized Beighton protocol using a goniometer, should be part of the assessment for children referred for motor coordination problems, since this may include a variable to consider in setting intervention goals on controlling and strengthening or stabilising movements.

It is well known that dealing with more degrees of freedom augments the complexity of motor control processes [20,21]. Moreover it could enlarge loads on postural control due to decreased joint stability. In particular, the increased knee extension should be taken into account from a postural control perspective. According to our findings this extension was significantly related to the MABC2 score (left knee r_s_ = −0.48, *p <* .01; right knee r_s_ = −0.37, *p* = .03).

Looking at children with DCD one wonders by what mechanism more mobility or larger degrees of freedom in a joint might co-occur with motor coordination problems. According to Maillard and Murray [11] the aspect of reduced proprioception from the joints in children with hypermobility might lead to poor control of joint movement and instability. According to Geuze [33] the major characteristics of poor control in DCD are inconsistent timing of muscle activation sequences, co-contraction and lack of automatization and slowness of response. These characteristics will make it more difficult to control hypermobile joints, since a lack of co-contraction and slowness of response will result in decreased and less well timed stability of the loaded joints. This current study supports the notion that having to deal with larger degrees of freedom in joints can co-occur with motor problems in children with DCD. During development children might find a way to strengthen and control their hypermobile joints. For hypermobile knees this idea is supported by the study of Greenwood et al. [34]. Electromyography showed significantly higher semitendinosus activation overall, and significantly higher co-contraction of Rectus femoris and Semitendinosus during less challenging tasks (two-leg standing) of hypermobile adult participants with JHS compared to a control group. In contrast it is also known that children with high motor proficiency may excel if they have mobile joints, likely because they can exploit the larger degrees of freedom by intensive training (e.g. gymnasts). It is obvious that the stability of a joint is not only dependent upon intact ligament structures but also on neuromuscular control and muscle tone [1].

The finding that the number of children that could get their hands on the floor with straight legs (*χ*^2^ = 1.86, *p* = .17) did not differ between the matched TD-group and DCD-group is of interest. This links to the findings of Cantell and Hands that increased flexibility is not found in children with poor motor performance while doing the sit and reach test [15,16]. The most probable explanation is that both the Beighton component of standing with straight legs and touching the ground with both hands flat on the floor, and the sit and reach test are muscle length tests of the Hamstrings and not range of motion tests of a joint. It may be worthwhile to evaluate the validity of this item of the Beighton as to whether it gives appropriate information on hypermobility.

The combination of generating appropriate levels of force and having to deal with more degrees of freedom in a joint may negatively affect motor performance in hypermobile children with DCD. Having to generate increased force in antagonistic muscles and more co-contraction may also cause extra recruitment noise and therefore cause additional movement variability, which is frequently reported in movement patterns of children with DCD [35,36]. This statement is supported by the findings of Smits-Engelsman et al. in which children with DCD could produce the same level of maximum finger force as typically developing children but have poor control over maintaining steady force levels as required in the force control tasks [37]. Such a lack of fine tuned force control will lead to larger errors in precise movements.

We realize the number of girls and boys was not totally matched in the matched TD group compared to the DCD group. Jansson et al. found in Sweden a significantly higher degree of general joint laxity in girls of all ages compared to boys (*p* < 0.05) [6]. In contrast, three other studies found no significant differences by gender. First Rikken-Bultman found no significant difference in the group of a primary school [5]. Secondly van der Giessen found no differences in the prevalence of both hypermobility and the connective tissue signs between boys and girls with *p* values ranging from 0.115 to 1.000 [25]. Thirdly Smits-Engelsman recorded no significant differences (*p* = .22) for sex and if analyzed per item, only hands on the floor (item 5) was different (t (1549) = 4.66, *p* < .001); with girls being more flexible than boys [24]. So we had no reason to analyze sex differences and it is unlikely that sex differences explain any of the group differences at this age (7–10 years old).

The number of tests in the analysis of the association between MABC and different ROM has to be considered. On the other hand, one of the two knee extension measures would survive a correction for multiple tests. The fact that the other is also significant at the 0.05 level is clearly not irrelevant. It therefore seems reasonable to just describe the association as ‘significant’.

It should be taken into account that the clinical sample is small and larger samples would have been favoured. Also it would be an advantage to evaluate motor performance in more depth than is possible with a standardized 8 item test, like the MABC. Obviously a disadvantage of cross-sectional and correlation studies is that it is impossible to infer the developmental aetiology of the association. Do children with poor motor coordination become more mobile because they lack stabilization of their joints and as a result they increase their mobility? (For instance through repetitive joint sprains or as a strategy to stabilize the joint). It is important to investigate whether these children may adopt an altered posture whereby they “rest” or “hang” on the hip capsule and hip ligaments rather than activating their Gluteus medius, which would f.e. cause pelvic obliquity and instability [3]. The same can be expected for the knee and hanging in knee ligaments rather than activating the Quadriceps, Hamstrings and Gastrocnemius. Is deficient coordination the eliciting factor for instance through delayed or inadequate sensori-motor loops? Or are there other mediating factors that induce the co-occurrence of hypermobility and DCD? Further prospective and intervention research should elucidate the possible relations between underlying factors.

## Conclusion

Hypermobility, scored according to the cut-off scores of the Beighton protocol, is a frequent phenomenon. It is even more frequent in children with DCD. Although in the general population motor performance and joint mobility as measured ROM by goniometry are not related, this is the case in children referred for DCD. Hypermobility of the knee extension seem to co-occur with a lower percentile of the MABC. We argue that coping with hypermobility or larger degrees of freedom may be a disadvantage when motor coordination is deficient.

Clinicians need to consider joint mobility, measured according to the Beighton protocol, as a factor in their assessment of children referred for coordination problems to find out whether hypermobility should be considered an implicating factor in children with poor motor control.

## Abbreviations

DCD: Developmental coordination disorder; JHS: Joint hypermobility syndrome; MABC: Movement assessment battery for children; TDC: Typically developing children.

## Competing interests

The authors declare that they have no competing interests.

## Authors’ contributions

DJ conceived of the study, developed the design, tested one third of the children and wrote the article. BS helped develop the design ideas, performed the statistical analysis and both she and RG helped to draft the manuscript. MK and AN participated in the development of the study and initially reviewed the article. All authors read and approved the final manuscript.

## Pre-publication history

The pre-publication history for this paper can be accessed here:

http://www.biomedcentral.com/1471-2431/13/35/prepub
